# Short-Term Ingestion of Medium-Chain Triglycerides Could Enhance Postprandial Consumption of Ingested Fat in Individuals with a Body Mass Index from 25 to Less than 30: A Randomized, Placebo-Controlled, Double-Blind Crossover Study

**DOI:** 10.3390/nu14051119

**Published:** 2022-03-07

**Authors:** Naohisa Nosaka, Shougo Tsujino, Kazuhiko Kato

**Affiliations:** 1 Central Research Laboratory, The Nisshin OilliO Group, Ltd., 1 Shinmori-Cho, Isogo-ku, Yokohama 235-8558, Japan; s-tsujino@nisshin-oillio.com; 2 Kato Clinic, 1-1-1 Nakaizumi, Komae 201-0012, Japan; kato-kaz@db3.so-net.ne.jp

**Keywords:** medium-chain triglycerides (MCTs), obesity, energy expenditure, diet-derived fat, postprandial resting metabolism, sedentary, octanoic acid, decanoic acid

## Abstract

The elimination of obesity is essential to maintaining good health. Medium-chain triglycerides (MCTs) inhibit fat accumulation. However, studies examining energy expenditure and fat oxidation with continuous ingestion of MCTs show little association with the elimination of obesity. In this study, we conducted a randomized, double-blind crossover clinical trial to investigate the effects of continuous ingestion of MCTs on postprandial energy expenditure and ingested long-chain triglycerides (LCTs) oxidation. A daily 2 g of MCTs were ingested for two weeks by sedentary participants with a body mass index (BMI) from 25 (kg/m^2^) to less than 30. Ingestion of a meal containing MCTs and isotopic carbon-13-labeled (13C) LCTs increased energy expenditure and consumption of diet-derived LCTs, as determined by postprandial 13C carbon dioxide excretion, compared to canola oil as the placebo control. These results indicate that continuous ingestion of MCTs could enhance postprandial degradation of diet-derived fat and energy expenditure in sedentary, overweight individuals.

## 1. Introduction

Obesity is well-known as a risk factor for metabolic diseases such as hypertension, dyslipidemia, and type 2 diabetes [1,2,3]. Its elimination is essential to maintaining good health by reducing the occurrence and extension of health problems caused by metabolic diseases [4]. The basic strategy to eliminate obesity is to increase energy expenditure and limit energy intake [4,5]. Lipids, one of the energy-producing nutrients, have been shown to have varying effects on obesity, depending on the types of fatty acids [6]. It is crucial to examine the effects of fatty acids in lipids on energy and lipid metabolism relating to obesity.

Medium-chain fatty acids (MCFAs) are widely defined as straight-chain saturated fatty acids with a chain length ranging from 6 to 12 carbon atoms [7,8]. In a nutritional study, research on the alternative use of long-chain fatty acids started mainly on fatty acids with a chain length of 8 carbon atoms (octanoic acid, C8) and 10 carbon atoms (decanoic acid, C10) [7,9]. This was followed by research on biological regulatory functions different from those of long-chain fatty acids, one of which was research on the elimination of obesity [10,11].

Continuous ingestion of medium-chain triglycerides (MCTs) or medium- and long-chain triglycerides (MLCTs) to suppress fat accumulation has been compared to ingestion of long-chain triglycerides (LCTs) [12,13,14,15,16]. Continuous ingestion of MCTs to enhance fat oxidation during low-intensity physical activity was reported over the effect of LCTs [7,17]. However, there are few results on the effects of short-term MCT ingestion on postprandial energy and lipid metabolism, which can be associated with the elimination of obesity. Studies examining postprandial enhancement of energy expenditure with continuous ingestion of MCTs have not reached a consensus; some reports [18,19,20] show a significant increase while others [21] find no difference. Studies examining the increase in fat oxidation after meals have reported either positive [19,20] or negative [18] effects. Ingested MCTs are oxidized more than LCTs [22,23]. However, little is known about the enhanced degradation of dietary LCTs when MCTs are ingested continuously.

In this study, we conducted a randomized, double-blind crossover clinical trial, using canola oil as a placebo control, to examine the effects of continuous ingestion of MCTs on postprandial ingested LCTs oxidation, energy expenditure, and fat oxidation. Individuals with a body mass index (BMI) over or equal to 25 (kg/m^2^) and less than 30 were given 2 g of MCTs daily for two weeks, followed by a meal containing MCTs and carbon 13 isotope-labeled (13C) LCTs. To measure 13C carbon dioxide, expired air was collected postprandially. Oxygen consumption and carbon dioxide production were measured to examine energy expenditure and fat oxidation after a meal.

## 2. Materials and Methods

Ethical considerations, setting the target number of participants, eligibility and exclusion criteria, the oils and fats studied, study design, participant compliance matters during the intervention period, and dietary surveys during the intervention period were briefly described. These details were detailed previously [7].

### 2.1. Ethical Considerations

The present study was conducted in compliance with the Declaration of Helsinki (revised in 2013), the Japanese Ethical Guidelines for Medical and Health Research Involving Human Subjects, and the Japanese Act on the Protection of Personal Information [24,25]. We obtained approval from the ethics committees of Yoga Allergy Clinic (approval number 21000023). We registered this clinical trial in UMIN-CTR before the recruitment of participants (UMIN000043022, URL: https://upload.umin.ac.jp/cgi-open-bin/ctr/ctr.cgi?function=brows&action=brows&recptno=R000049074&type=summary&language=E, accessed on 24 January 2022).

### 2.2. The Number of Participants

The target number of participants was set at 30. The number of participants required was estimated to be a minimum of 18 or 23 based on the evaluation indicators of a previous study [17]. The number of participants was determined by comparing the MCT intake and measuring instrument of a previous study with the present study.

### 2.3. Target Participants

The target participants were those who met the following criteria: healthy Japanese males and females aged between 35 and 64 years (at the time of obtaining written consent); a BMI over or equal to 25 and less than 30; non-smokers; consumed less than 30 g/day of alcohol; those who received a sufficient explanation of the purpose and content of the research, had the capacity to consent, had volunteered willingly to participate in the study, had a good understanding of the study, and agreed to participate in the study in writing.

Those who met the following criteria were excluded from the participants: persons with serious diseases or histories (cardiac, hepatic, renal, cardiovascular, or hematological diseases); who had experienced chest pain or abnormal pulse while at rest; who were currently undergoing exercise or diet therapy under the medical supervision of a physician; who frequently experienced shortness of breath, dizziness, vertigo, or loss of consciousness; who were allergic to drugs, food, raw materials of test meal (soybeans, milk protein), or had a history of allergy; who had a current or past history of drug or alcohol dependence; who take any health foods, supplements, or drugs that may affect fatigue reduction, fat burning, or obesity control; who exercised to maintain or improve physical fitness for a total of 60 min or more per week; whose weight fluctuated by ±5 kg or more over two months.

### 2.4. Test Diets

MCTs (The Nisshin OilliO Group, Ltd., Tokyo, Japan) (C8:C10 ratio = 75:25) was used as the test oil (TO). The control oil (CO) was canola oil, a rapeseed oil with low erucic acid (The Nisshin OilliO Group, Ltd., Tokyo, Japan). The participants consumed the TO or the CO at 2 g per day for the 13-day intervention periods.

### 2.5. Management during the Intervention Period

The present study consisted of two 13-day interventions and a one-day measurement, separated by a 14-day washout period. We asked the participants to maintain body weight; consume the TO or CO; weigh themselves daily; make as few changes as possible to their lifestyle and environment (such as meals, alcohol consumption [less than 30 g/day], exercise, sleep, and work); not ingest any medicines, supplements, health foods (including oil for health), or functional foods that may affect the reduction of fatigue, fat burning, or obesity, and report the results daily for electronic recording; refrain from strenuous exercise for three days before the measurement; and refrain from drinking alcohol on the day before that.

### 2.6. Dietary Survey

For three days, the 11th to 13th days after TO or CO ingestion, we asked the participants to take photographs of their meals and complete record sheets. We calculated nutritional values from the photos and record sheets, and determined the daily intake of energy, protein, fat, carbohydrate, C8, and C10. In calculating nutritional values, we used the Standard Tables of Food Composition in Japan 2015 (seventh revised version) [26].

### 2.7. Measurements

We used an ordinary pressure-type human calorimeter (Fuji Human Calorimeter FHC-30S, Fuji Medical Science Co., Ltd., Chiba, Japan) installed in the metabolic measurement room. The specification of the human calorimeter was described previously [7]. The inflow rate of air into the room was set at 50 L/min, and the air supply and exhaust were controlled at the same rate with an accuracy of ±0.5%. The room temperature and humidity were controlled at 25 °C ± 0.1 °C and 50% ± 1.0%, respectively. The accuracy of an analyzer (Thermo Scientific Prima Pro, Process Mass Spectrometer, Thermo Fisher Scientific, Inc., Waltham, MA, USA) installed in the human calorimeter for the exhausted oxygen and carbon dioxide was ±0.002%. The accuracy of the measurements during this study was confirmed by conducting three alcohol combustion tests before, during, and after the study. The theoretical values of oxygen consumption and carbon dioxide production were calculated from the weight of alcohol burned. These measured values were obtained by analyzing the measured data using Henning’s formula [27]. The recovery rate (formula: (measured value/theoretical value) × 100; unit: %), which is the measurement accuracy expressed from the theoretical and measured values, was within 100% ± 2% for both oxygen consumption and carbon dioxide production.

The participants visited the metabolic measurement room in an overnight fasted state and consumed a meal (energy 483 kcal, protein 15.5 g, fat 15.2 g, carbohydrate 75.1 g) containing 2 g of TO or CO and 400 mg of 13C triolein (TRIOLEIN, 1,1,1-13C3, purity 99%, CLM-163-PK, Cambridge Isotope Laboratories, Inc., Tewksbury, MA, USA).

The resting oxygen uptake rate and carbon dioxide production rate were measured with participants in a sitting position, over a period of time from before meal ingestion to four hours afterwards. The measured values were analyzed using Henning’s formula. Expired gas was collected every hour, using a gas bag (Tedlar bag, 1-2711-04, As One Corporation, Osaka, Japan). The collected expired gas was used to measure the carbon 13/carbon 12 ratio in carbon dioxide, using the gas analyzer installed in the human calorimeter.

### 2.8. Calculation of the Consumption Rate of Diet-Derived LCTs

To determine the consumption rate of diet-derived LCTs, we used the following equation to calculate the consumption rate of 13C triolein, based on the carbon 13/carbon 12 ratio in carbon dioxide of the collected expired gas, the carbon dioxide production rate obtained from the human calorimeter, and the ingested 13C triolein weight [28].

Consumption rate of diet-derived LCTs (%) = {(carbon 13/carbon 12 ratio in carbon dioxide after ingestion − carbon 13/carbon 12 ratio in carbon dioxide before ingestion) × carbon dioxide production rate after ingestion × 100}/{ingested weight of 13C triolein × (number of carbon 13 labels in 13C triolein/molecular weight of 13C triolein) × 22.4}.

To determine the cumulative consumption rate of postprandial diet-derived LCTs from the obtained consumption rate of diet-derived LCTs over time, the area under the curve (AUC) was determined using the trapezoidal method.

### 2.9. Calculation of Postprandial Energy Expenditure Rate, Respiratory Quotient, and Fat and Carbohydrate Oxidation Rates

We calculated the respiratory quotient (RQ), fat oxidation rate, carbohydrate oxidation rate, and energy expenditure rate from the oxygen uptake rate and carbon dioxide production rate obtained from the human calorimeter, using the following equations [29].

RQ = carbon dioxide production rate/oxygen uptake rate.

Fat oxidation rate (mg/min) = 1.67 × oxygen uptake rate − 1.67 × carbon dioxide production rate.

Carbohydrate oxidation rate (mg/min) = 4.55 × carbon dioxide production rate − 3.21 × oxygen uptake rate.

Energy expenditure rate (kcal/min) = 9.75 × fat oxidation rate + 3.74 × carbohydrate oxidation rate.

We calculated the AUC using the trapezoidal method to determine the cumulative value of energy expenditure, fat and carbohydrate oxidation from the energy expenditure rate, and rates of fat and carbohydrate oxidation measured over time.

### 2.10. Primary and Secondary Outcomes

The primary outcome was the cumulative consumption rate of diet-derived LCTs. Secondary outcomes were cumulative values of energy expenditure, fat and carbohydrate oxidation, energy expenditure rate, and RQ.

### 2.11. Statistical Analysis

The cumulative consumption rate of diet-derived LCTs and the cumulative values of energy expenditure and fat and carbohydrate oxidation during a four-hour postprandial period were checked for normality by a Shapiro–Wilk test, to compare between the TO and CO intervention groups. If there was no normality, we performed a Mann–Whitney U test. If there was normality, we used the F test to check for equivariance. We conducted a Student’s *t*-test if there was an equivariance, and a Welch’s *t*-test if equivariance was absent. We analyzed for significance the intervention effect (the value of the TO group minus that of the CO group) of the cumulative consumption rate of diet-derived LCTs and cumulative values of energy expenditure and fat and carbohydrate oxidation during the four-hour postprandial period, using the Mann–Whitney U test.

In the difference of postprandial longitudinal energy expenditure and RQ, and their intervention effect in the TO and CO groups, we compared data using a linear mixed model. We set the intervention group, time, and ingestion order as a fixed effect, and the participant as a random effect. We estimated the values by the restricted maximum likelihood method with a random intercept model equation. We analyzed the fixed effect for significance and determined estimates and 95% confidence intervals. If the estimated values for the intervention group were significant, normality was checked with the Shapiro–Wilk test to compare the values for each time between the two intervention groups. If there was no normality, a Mann–Whitney U test was conducted. If there was normality, we conducted an F test to check for equivariance. We performed a Student’s *t*-test if equivariance was present, and a Welch’s *t*-test if equivariance was not present.

When measurement indices showed significant differences, we analyzed carryover effects. If we found them to be of significance, we withheld the results.

We used Microsoft Excel for Office365 MSO (Microsoft Japan Co., Ltd., Tokyo, Japan) to calculate the basic statistics of the analyzed data. We used the statistical package R (Version 3.4.3, R Core Team, Vienna, Austria) for statistical processing. For all tests, *p* values of less than 5% were considered to indicate a significant difference.

## 3. Results

Some of the data (metabolic data during low-intensity physical activity) obtained in this study has been published previously [7]. An analysis of metabolic data during resting was conducted in this study.

### 3.1. Participants

Seventy-two participants who obtained written informed consent were screened by interviews, physical measurements, and biochemical and hematological tests. Thirty eligible participants were randomly assigned to two groups (14 participants and 16 participants)—they ingested the test diets and were measured. One participant discontinued the study before the second measurement. Twenty-nine participants completed the study. A flowchart of the participants was provided previously [7]. Those 29 participants were used as the study participants, for analysis. The 29 analysis participants (17 males and 13 females) at the screening were aged 50.3 ± 9.1 (mean ± standard deviation), 165.4 ± 8.9 cm height, 73.9 ± 9.5 kg weight, and BMI 26.8 ± 1.3 kg/m^2^.

### 3.2. Dietary Intake

There were no significant differences in energy (CO: 1798.2 ± 316.1; TO: 1974.8 ± 359.4 kcal, mean ± standard deviation), protein (CO: 67.2 ± 14.2; TO: 71.3 ± 16.5 g), carbohydrate (CO: 226.5 ± 61.8; TO: 246.2 ± 57.3 g), and fat (CO: 64.8 ± 15.6; TO: 73.7 ± 22.6 g) intakes between the two groups. Intakes of MCFAs (C8, C10) were significantly greater in the TO group (C8: 2.0 ± 0.1; C10: 0.9 ± 0.2 g) than in the CO group (C8: 0.1 ± 0.1; C10: 0.2 ± 0.2 g).

### 3.3. Measurement Result of Primary Outcome

The intervention effect value in the cumulative consumption rate of diet-derived LCTs was 0.7% ± 0.4% (mean ± standard error), which indicated a significantly greater consumption rate in the TO group than in the CO group (Table 1).

### 3.4. Measurement Results of Secondary Outcomes

The cumulative value of energy expenditure in the TO group was 27.0 ± 2.5 kcal, which was significantly greater than that in the CO group (19.5 ± 2.0 kcal). As for the cumulative values of fat and carbohydrate oxidation, there were no significant differences between the two groups (Table 1).

Changes in measurement values of energy expenditure rate and RQ were not significantly different between the two groups (Table 2).

### 3.5. Carryover Effect of Measurement Outcomes

There were no significant carryover effects in the measurement outcomes.

## 4. Discussion

In a survey of the Japanese population, approximately 30% of men and 20% of women had a BMI of 25 or higher [30]. The overweight participants in the present study, with a BMI over or equal to 25 and less than 30, are classified as pre-obese by the World Health Organization [31] and obese level I in Japan.

In this study, we investigated the beneficial effect of MCTs on postprandial ingested fat consumption, which could be involved in the elimination of obesity. Stable isotope-labeled LCTs were fed to the participants. We measured the isotope-labeled carbon dioxide production associated with the increased degradation of diet-derived LCTs. The results showed that the continuous ingestion of MCTs significantly enhanced postprandial consumption of diet-derived fat, compared to canola oil. The intake of MCFAs (C8 and C10) by the CO group during the two-week intervention period was equivalent to approximately one-tenth that of the TO group, which approximates to the average intake of Japanese people. Estimating from the report of the National Health and Nutrition Examination Survey in Japan [30], MEXT meeting materials [32], and the Standard Tables of Food Composition in Japan 2015 (seventh revised edition) [26], the average total C8 and C10 intake by Japanese people is 0.2–0.3 g (2003: 0.252 g; 2008: 0.230 g; 2013: 0.249 g; 2018: 0.265 g; 2019: 0.271 g), and the percentage of total fat intake is approximately 0.5% (2003: 0.528%; 2008: 0.499%; 2013: 0.512%; 2018: 0.496%; 2019: 0.499%). The present study shows that continuous ingestion of 10 times more MCFAs (C8 and C10) than the daily intake by sedentary, overweight individuals may enhance the degradation of exogenous fat associated with the elimination of obesity to a greater extent than routinely consumed LCTs.

A previous study using isotope-labeled MCTs and LCTs showed that ingested MCTs are more degraded postprandially than ingested LCTs [22,23]. However, no studies seem to have reported accelerated degradation of ingested LCTs after a meal following continuous ingestion of MCTs. A previous study [33] examined the increased degradation of exogenous LCTs when continuously ingesting coconut oil (mainly lauric acid (C12) and myristic acid (C14)) as MCTs, which differs from the MCTs (C8:C10 = 75:25) in the present study. It reported that there was no difference or a decrease in the consumption of diet-derived LCTs, compared to continuous ingestion of beef tallow as the control diet.

Two previous studies examining the degradation of diet-derived 13C-triolein showed that it did not change in normal weight subjects (in a study examining differences in exercise habits [34]), and decreased in obese subjects, compared to normal weight subjects (in a study examining differences in a BMI [23]). Although a previous study suggested that the degradation of dietary triolein decreases with obesity, as shown in the present study, a two-week ingestion of MCTs significantly increased the consumption of diet-derived triolein in sedentary, overweight individuals, and short-term ingestion of MCTs could increase diet-derived fat degradation.

Multiple factors are involved in the degradation of LCTs after a meal: degradation by digestive enzymes in the intestinal tract, absorption from the intestinal tract, re-esterification and lipoprotein synthesis in small intestinal cells, lymphatic transport, lipoprotein metabolism in the blood, binding of free fatty acids to albumin, and metabolism of fatty acids taken up by organs [35]. Among these factors, fatty acid metabolism in the liver is considered possibly to affect the accelerated degradation of ingested LCTs by continuous ingestion of MCTs. LCTs in lipoproteins released into the blood after a meal are converted into fatty acids by lipoprotein lipase in the blood and distributed to adipose tissue, muscle tissue, and liver, which are the major organs that use fatty acids in the body [35,36]. Studies of animals have shown that consumption of MCFAs (C8 and C10) increases the degradation of fatty acids, including beta-oxidation, and increases the degradation of stored fat and the release of fatty acids into the bloodstream in adipose tissues [37,38,39]. Of these organs, the liver actively consumes energy even during resting [40], and ingested long-chain fatty acids from the diet that reach the liver after a meal may also accelerate their degradation.

In this study, a two-week ingestion of MCTs increased postprandial energy expenditure more than canola oil ingestion, in participants with an average BMI of 26.8. This suggests that continuous ingestion of MCTs by overweight individuals may increase postprandial energy expenditure, contributing to the resolution of obesity. Many clinical studies have reported increases in postprandial energy expenditure with single or multiple ingestions per day of MCTs [19,41,42,43] or MLCTs [44,45]. In a study of lean and obese subjects, postprandial energy expenditure was significantly higher when they consumed MCTs than when they consumed LCTs [46]. A negative result [21] in subjects with a mean BMI of 21.4 and a positive result [18] in subjects with a mean BMI of 22.6 were reported, for increased energy expenditure after a meal containing MCTs and after continuous ingestion of MCTs, with inconsistent results shown in lean individuals. On the other hand, positive results in subjects with mean BMIs of 26 and 31.8 have been reported [19,20]. In studies of pre-obese and obese individuals, the increase in energy expenditure after the meal containing MCTs and with continuously consumed MCTs was consistent, supporting the results of the present study.

White et al. [21] showed negative results in enhancing the effect of continuous ingestion of MCTs on energy expenditure, pointing out that a higher proportion of MCTs in the fat ingested to evaluate energy expenditure may increase energy expenditure. They used butter and coconut oil as sources of MCFAs, and the fats in the evaluation had 7.9% MCFAs (C8 and C10) in total fatty acids. Compared to the control, C12 and C14 were approximately 16% and 9% higher, and oleic acid was approximately 15% lower. In the evaluation diet of the present study, MCTs were approximately 13% in total fat and did not differ, except for the 2 g of TO and CO. In previous single-dose studies with relatively low intakes of MCFAs (C8 and C10), the C8 and C10 in the fat were approximately 4% when the results were negative for effects on postprandial energy expenditure [47], whereas the C8 and C10 in the diet were approximately 12% when the results were positive [45]. Although the proportion of MCTs in fat intake that shows an effect of increased energy expenditure after a meal is not sufficiently clear, the ratio may affect energy expenditure after a meal.

In this study, fat and carbohydrate oxidation did not differ from the CO group in substrate utilization, when the significant increase in postprandial energy expenditure occurred after consumption of MCTs in the TO group. There was no significant change in body weight during the intervention period. The amount of energy intake was within the range of the daily amount of the participants, and the fat energy intake was approximately 33%, which is similar to the daily diet of Japanese people. A previous human study reported that fat oxidation did not increase when the subjects consumed 15 g of MCTs daily, within the daily energy intake range, and less than 30% of fat energy [18]. Studies of animals have reported that consumption of MCTs enhances carbohydrate utilization. Continuous ingestion of MCTs resulted in increased glucose uptake in muscle tissue and de novo fatty acid synthesis, resulting in increased glucose uptake in the liver [48,49]. These studies indicate that continuous ingestion of MCTs may enhance the utilization of both carbohydrate and fat when postprandial energy expenditure is increased. On the other hand, one human study with 150% of energy intake of daily energy [19] and another with 40% of fat energy during the interventions [20] reported that fat oxidation during continuous ingestion of MCTs is higher than for ingestion of LCTs. One possible cause of this difference in results is an increase in the energy proportion of fat to the total energy. In a study that measured 24 h RQ after two days of ingesting diets with 20% and 50% fat energy, RQ was significantly lower and fat oxidation was higher in both lean and obese subjects after consuming a diet with 50% fat energy [34]. In a study of individuals with an average BMI of 24, a three-day high-fat diet (40% fat energy) resulted in more degradation of the ingested fat than a high-carbohydrate diet (10% fat energy) [50]. Even in a single-dose study, the rate of fat oxidation after a fat-rich diet increased significantly, compared to a carbohydrate-rich diet, in both lean and obese individuals [51]. These studies suggest that postprandial fat oxidation may have more to do with the ratio of fat energy in total energy than with differences in fatty acids of the lipid (MCTs or LCTs). In the present study, RQ did not decrease after a meal containing MCTs when the ratio of fat energy to total energy was approximately 33%. Continuous ingestion of MCTs enhances postprandial energy expenditure and may lead to the elimination of obesity, but the substrates used for energy expenditure may depend on the balance between fat and carbohydrate intakes.

On the other hand, continuous ingestion of MCTs could enhance lipolysis when there is increased energy expenditure in the skeletal muscle and may contribute to the resolution of obesity. In reported human studies, continuous ingestion of MCTs increased fat oxidation during aerobic exercise in recreational athletes [52] and people without exercise habits [7,17], compared to ingestion of LCTs. Studies of animals have reported explainable action mechanisms for increased fat oxidation during aerobic exercise, including increased mitochondrial biosynthesis in skeletal muscle, increased fat transport, and increased ketone body metabolism [49,53,54]. In addition, continuous ingestion of MCTs and MLCTs has been reported to increase fat metabolism, which may contribute to the resolution of obesity. Studies of animals have shown increased expression of uncoupling protein 1 (in adipose tissue) [39] and uncoupling protein 3 (in the mitochondria of skeletal muscle) [38], and increased enzyme activities and expression of genes involved in fatty acid degradation in the liver [37]. It has been shown that continuous ingestion of MCTs may lead to the elimination of obesity, with various responses that differ from those of ingestion of LCTs when the metabolism of energy-producing nutrients is dramatically changed, such as through diet or physical activity.

The limitations of the present study are the following. First, although we observed a positive effect on postprandial ingested fat consumption after the two-week intervention period, the direct effect on the accumulation of body fat is unknown. Second, although we observed a significant increase in energy expenditure in the four hours after a meal, the daily energy expenditure is unknown. Third, although we observed a positive effect of MCTs on the daily diet of the Japanese, the effects of MCTs when consuming diets of different compositions of macro-nutrients (protein, fat, and carbohydrate) is unknown.

## 5. Conclusions

From the results of the present study, continuous ingestion of 2 g/day of MCTs by individuals with a BMI over or equal to 25 and less than 30 kg/m^2^ could enhance the oxidation of diet-derived LCTs. The mechanism of action may be enhanced fatty acid degradation in the liver, caused by the continuous ingestion of MCTs. In addition, continuous ingestion of MCTs could increase postprandial energy expenditure (compared to canola oil) in sedentary, overweight individuals, but the substrates consumed for energy may depend on the ratios of fat and carbohydrate to total energy intake.

## Figures and Tables

**Table 1:** Cumulative values for four hours after a meal ^1^ Cumulative consumption rate of diet-derived LCTs and cumulative values of energy expenditure and fat and carbohydrate oxidation in the postprandial measurement.

	CO Group	TO Group	Intervention Effect Value (TO–CO)
Rate of diet-derived LCTs, %	3.8 ± 0.5	4.5 ± 0.5	0.7 ± 0.4 ^#^
Energy expenditure, kcal	18.6 ± 2.1	26.1 ± 2.6 *	7.5 ± 3.3
Fat oxidation, g	0.6 ± 0.3	0.7 ± 0.3	0.1 ± 0.2
Carbohydrate oxidation, g	19.1 ± 1.9	18.5 ± 1.8	−0.7 ± 2.3

^1^ Values are expressed as means ± standard errors. *n* = 29. * Significant difference compared to control group (*p* < 0.05, Mann–Whitney U test). ^#^ Significant mean difference was detected (*p* < 0.05, Mann–Whitney U test).

**Table 2:** Changes in measurement values over time for four hours after a meal ^1^; energy expenditure rate and respiratory quotient in the postprandial measurement.

	CO Group	TO Group	Intervention Effect Value (TO–CO)
Energy expenditure rate, kcal/min			
Baseline	1.12 ± 0.03	1.10 ± 0.03	−0.021 ± 0.014
1 h after	1.25 ± 0.04	1.26 ± 0.04	0.016 ± 0.015
2 h after	1.23 ± 0.03	1.25 ± 0.03	0.021 ± 0.017
3 h after	1.14 ± 0.03	1.15 ± 0.03	0.014 ± 0.012
4 h after	1.09 ± 0.03	1.10 ± 0.03	0.005 ± 0.013
Respiratory quotient			
Baseline	0.81 ± 0.01	0.82 ± 0.01	0.013 ± 0.011
1 h after	0.87 ± 0.01	0.88 ± 0.01	0.008 ± 0.007
2 h after	0.89 ± 0.01	0.89 ± 0.01	−0.001 ± 0.012
3 h after	0.90 ± 0.01	0.89 ± 0.01	−0.010 ± 0.011
4 h after	0.85 ± 0.01	0.86 ± 0.01	0.014 ± 0.009

^1^ Values are expressed as means ± standard errors. *n* = 29. There were no significant differences between the groups.

## Data Availability

Data not available due to commercial restrictions.
